# Six Years Observation After Successful Treatment of Bacterial Vaginosis

**DOI:** 10.1155/S1064744997000513

**Published:** 1997

**Authors:** Jane Boris, Carl Påhlson, P.-G. Larsson

**Affiliations:** 1 Department of Obstetrics and Gynecology Kärnsjukhuset Skövde S-541 85 Sweden; 2 Department of Infectious Diseases and Clinical Microbiology University of Uppsala Uppsala Sweden

## Abstract

*Objective:* The cure rate after treatment of bacterial vaginosis (BV) differs in various investigations, but most studies report a cure rate of 70% after 1 month.

*Methods:* A long-term observation study after successful treatment of BV has been undertaken. The original study was a treatment study of BV and included 50 patients.

*Results:* We were able to identify 44 of the original 50 patients. The mean follow-up time was 6.9 years (range 4.7–9 years). During this time, 21 women (48%) had been free of BV while 23 women had had relapses. There was no difference in the use of broad-spectrum antibiotics, episodes of candida vaginitis, bleeding disturbances, family planning method, development of cervical intraepithelial neoplasia (CIN), or gynecological operations between women with and without relapses. The women with relapses had had a new sexual contact more often during the observation period than women without relapses. There was no difference in hydrogen peroxide production of the lactobacilli among women with or without relapses, and survival analysis shows that most relapses occur during the first year after treatment.

*Conclusions:* If patients are successfully treated, half of the patients will stay cured indicating that treatment is of benefit. Most relapses occur during the first year. Our results indicate that the etiology of BV might have something to do with new sexual contacts.


Infectious Diseases in Obstetrics and Gynecology 5:297–302 (1997)
© 1998 Wiley-Liss, Inc.

### Six Years Observation After Successful Treatment of Bacterial Vaginosis

**Jane Boris,[1]* Carl Påhlson,[2] and P.-G. Larsson[1]**

[1]*Department of Obstetrics and Gynecology, Kärnsjukhuset, Skövde, Sweden*
[2]*Department of Infectious Diseases and Clinical Microbiology, University of Uppsala, Uppsala, Sweden*

### Abstract

***Objective:*** **The cure rate after treatment of bacterial vaginosis (BV) differs in various investigations, but most studies report a cure rate of 70% after 1 month.**

***Methods:*** **A long-term observation study after successful treatment of BV has been undertaken. The original study was a treatment study of BV and included 50 patients.**

***Results:*** **We were able to identify 44 of the original 50 patients. The mean follow-up time was 6.9 years (range 4.7–9 years). During this time, 21 women (48%) had been free of BV while 23 women had had relapses. There was no difference in the use of broad-spectrum antibiotics, episodes of candida vaginitis, bleeding disturbances, family planning method, development of cervical intraepithelial neoplasia (CIN), or gynecological operations between women with and without relapses. The women with relapses had had a new sexual contact more often during the observation period than women without relapses. There was no difference in hydrogen peroxide production of the lactobacilli among women with or without relapses, and survival analysis shows that most relapses occur during the first year after treatment.**

***Conclusions:*** **If patients are successfully treated, half of the patients will stay cured indicating that treatment is of benefit. Most relapses occur during the first year. Our results indicate that the etiology of BV might have something to do with new sexual contacts. Infect. Dis. Obstet. Gynecol. 5:297–302, 1997.** © 1998 Wiley-Liss, Inc.

Key words follow-up; vaginitis; metronidazole; lactobacilli

The cure rates after treatment of bacterial vaginosis (BV) differ in various studies. The drug of choice has been metronidazole tablets 500 mg BID for 7 days.[1] The 1 week cure rate after start of treatment is said to be about 90%, while evaluation after 1 month reveals a lower cure rate. In open studies the 1 month cure rate is 82%, while in blind studies it is 65%. In the few published studies with follow-up time of 3 months or more the cure rate ranged from 25 to 77%.[2,3] No treatment studies have been published with follow-up times longer than 6 months.[4]

Several factors could influence the rate of recurrent BV: 1) inadequately treated infection, 2) the patient's local defense system, partly represented by the $H_2O_2$-producing lactobacilli in the vagina, 3) other vaginal infections, or 4) the consumption of antibiotics. For sexually transmitted diseases (STDs) the number of sexual partners is known to affect the relapse rate, but it is still controversial

Contract grant sponsor: Swedish Society of Medicine (SLS); Contract grant number: 92; Contract grant sponsor: Skaraborg County Development Fund; Contract grant number: 177/92.

*Correspondence to: Dr. Jane Boris, Department of Obstetrics and Gynecology, Kärnsjukhuset, S-541 85 Skövde, Sweden. E-mail: p.g.larsson@ltskar.se

Received 12 May 1997
Accepted 29 August 1997

Clinical Study whether BV should be regarded as an STD. Hart[5] found BV to be associated with sexual activity but not other STDs and among pregnant women there is shown to be a coinfection between BV and other STDs.[6] However, in epidemiological studies among non-pregnant women, BV has not been shown to be an STD,[7,8] and partner treatment has not been shown to be of any benefit in the cure rate.[2]

The purpose of this study was to evaluate the long-term cure rate after successful treatment of BV, to retrospectively register the number of retreatments of BV during the study period, and to evaluate the factors that could possibly predict relapses of BV.

### SUBJECTS AND METHODS

### Patients

This study is based upon a previously performed double blind, placebo controlled treatment study of metronidazole 500 mg TID for 10 days.[4] The inclusion criteria for the initial study were fulfilling three of four criteria for BV set by Amsel et al.[9] (typical discharge, elevated pH, positive sniff test, and presence of clue cells in wet smear). To this an additional criterion was added: the motile rod-shaped bacteria *Mobiluncus* should be seen by wet smear microscopy.

In the primary study, 50 patients were enrolled. All patients treated with placebo or those with treatment failure after 1 month were retreated with the same regimen of metronidazole tablets and once again followed up 1 month later until they were cured. A 6 month follow-up after successful treatment was performed. All further visits to our outpatient clinic after the 6 month follow-up were registered, and after about 5 years the patients were contacted and offered a study visit at our outpatient clinic. Women that had moved from our area were traced through their security number. The women had a personal interview and answered a preformed questionnaire, were examined for BV, and had a swab taken for culture of lactobacilli. Women who were not able to come to our clinic were sent a written instruction and a vaginal speculum to take a vaginal air-dried smear themselves and to take a vaginal sample with a cotton swab which was sent for culture. Smears that are obtained blindly have been shown to be reliable for the diagnosis of BV.[10] The air-dried smears taken outside our clinic were sent to us by mail, thereby making it possible for us to examine all of the smears ourselves according to a previously described method.[11]

### Microbiological Examination

Vaginal samples were collected by cotton swabs and transported by mail in Stuart's medium to the laboratory. Cultures for lactobacilli were performed anaerobically on Rogosa agar plates and blood agar plates. Genus and species determinations of isolated gram-positive, catalase-negative rods were performed by biochemical methods (API-Coryne, Merieux, France) and gas chromatography and supplemented by DNA sequencing of the gene for 16S rRNA. Isolated strains were tested for hydrogen peroxide production by the method described by Eschenbach et al.[12] If more than one lactobacillus strain was isolated, but only one was $H_2O_2$-producing, we assumed that the woman had an $H_2O_2$-producing strain of lactobacillus.

At the follow-up examination BV was diagnosed according to the established criteria of Amsel et al.[9] For the 4 patients with self-taken smears, the diagnosis was made solely by investigating the rehydrated air-dried smear. BV was then defined as presence of clue cells, overgrowth of coccoid-like bacteria, and lack of lactobacilli.[11]

### Questionnaire

All of the women answered a preformed questionnaire where we asked about intercurrent diseases since the initial study, surgery, pregnancies, family planning methods, bleeding disturbances, new sexual contacts, number of episodes of candida vaginitis, and use of antibiotics during the follow-up period.

Bleeding disturbances were defined as small intermenstrual bleeding episodes, spottings, or increased menstrual bleedings. Information about new sexual contacts was extracted from the questionnaire. Episodes of candida vaginitis were defined as candidiasis diagnosed and treated by a physician. During the study period it was not possible to buy anti-candida medications over the counter in Sweden. Nearly all patients had used some kind of antibiotics during the follow-up period. In case the patient had been treated for infections other than upper respiratory tract infections or lower urinary tract infections we presumed

TABLE 1. Number of relapses of BV during the observation period

| No. of relapses | No. of patients (n = 44) | Cumulative number of relapses |
|---|---|---|
| 0 | 21 (48%) | 0 (0%) |
| 1 | 11 (25%) | 11 (48%) |
| 2 | 7 (16%) | 18 (78%) |
| 3 | 4 (9%) | 22 (96%) |
| 4 | 1 (2%) | 23 (100%) |

that broad-spectrum antibiotics had been used. Number of relapses of BV during the observation period was recorded according to the patient's medical history, and both the diagnosis and treatment regimen were verified through the patient's medical record. The population in our community is rather stabile and there has not been any other gynecological outpatient clinic in the town until recently.

### Statistics

We used chi-square as statistics and $P < 0.05$ was defined as significant. The time from the initial proven cure to the first relapse, as well as the time from proven cure after the first relapse to the second relapse, were used in a survival analysis model.

### RESULTS

Of the original 50 patients, 6 women were not located because of emigration or a failure to answer our letter. The 44 remaining patients had a mean follow-up time of 6.9 years (range 4.7–9 years) since the successful completion of treatment for BV. Of the 44 patients, 21 had had no symptoms or signs of BV during the total study time. The other 23 patients had 1–4 diagnosed relapses of BV per patient (mean incidence of 1.8 per patient) (Table 1).

The mean age did not differ between women with (43.5 years) and without (43.8 years) relapse of BV. Significantly more women with relapse of BV reported new sexual contacts during the study period (Table 2). No differences were seen during the observation period among the two groups according to use of contraception, episodes of candida infections, treatment with broad-spectrum antibiotics, bleeding disturbances, cervical intraepithelial neoplasia (CIN), or gynecological diseases requiring operative intervention.

The time between initial proven cure and the first relapse is shown in Figure 1 using a survival analysis model. The first curve is based on 43 patients with 56 months of follow-up. One woman is not included in the survival curve as we were not able to identify the date of the first relapse. The second curve is based on 31 patients with 72 months of follow-up. Most of the women (16/22, 73%) relapsed during the first year after the initial treatment. We divided the relapses into early and late relapses (relapse within or after the first year of effective treatment). Five of 6 women (83%) with a late relapse of BV had a new sexual contact, compared to 63% (10/16) of the women with an early relapse ($P = 0.70$).

Six of 8 women who in the initial study needed a repeated metronidazole treatment for cure of BV had a new relapse of BV during the 6 years observation. Most of these relapses occurred as an early relapse (5/6).

We were able to culture *Lactobacillus* spp. from 22 of the 41 women with lactobacillus morphotype in the wet smear. Of these, 3 strains died before testing for $H_2O_2$ production. There was no difference in the isolation of $H_2O_2$-producing strains of lactobacilli among women with and without relapse. $H_2O_2$ production was present in 4 of 9 (44%) lactobacilli strains in the relapse group and 4 of 10 (40%) in the group who remained free of disease. In most cases, the lactobacilli were phenotypically classified into *Lactobacillus* spp. and no particular strain was correlated to women with relapse of BV. The genotypic classification showed that most strains were *L. acidophilus* and *L. plantarum*, and no difference between the women with and without relapses was found.

### DISCUSSION

The recurrence of BV after treatment may be high.[3] This has been used as an argument not to recommend the treatment of BV among asymptomatic women. Our results indicate that approximately half of the patients will remain free of BV for up to 5 years after initial successful treatment, indicating a benefit of treating women with BV.

BV is microbiologically characterized by a depletion of lactobacilli and overgrowth of *G. vaginalis* and anaerobes. The use of antibiotics could theoretically facilitate a bacterial shift toward a BV associated flora. In our study, all but two of the patients had used at least one treatment of antibiotics during the follow-up time. We divided the antibi-

TABLE 2. Differences between the relapse group and the women free of disease according to the results in the questionnaire

| | Free of disease (n = 21) | | Relapse group (n = 23) | | | |
|---|---|---|---|---|---|---|
| | % | No. | % | No. | *P* | Missing data |
| IUD use | 19 | (4) | 30 | (7) | NS | |
| Hormonal contraception | 29 | (6) | 26 | (6) | NS | |
| Candida infection | 43 | (9) | 52 | (12) | NS | |
| Broad-spectrum antibiotics | 47 | (8/17) | 53 | (7/13) | NS | 14[a] |
| Cured at first treatment | 95 | (19/20) | 72 | (16/22) | NS | 2[b] |
| Bleeding disturbances | 19 | (4) | 35 | (8) | NS | |
| CIN | 24 | (5) | 17 | (4) | NS | |
| Major gynecological surgery | 35 | (8) | 30 | (7) | NS | |
| Hysterectomy | 24 | (5) | 13 | (3) | NS | |
| Pregnancy | 9 | (2) | 18 | (4) | NS | |
| New sexual contact | 33 | (7) | 70 | (16) | <0.05 | |

[a]See text.
[b]Two patients were excluded because of treatment with other antibiotics for cervical *Chlamydia trachomatis* infection.

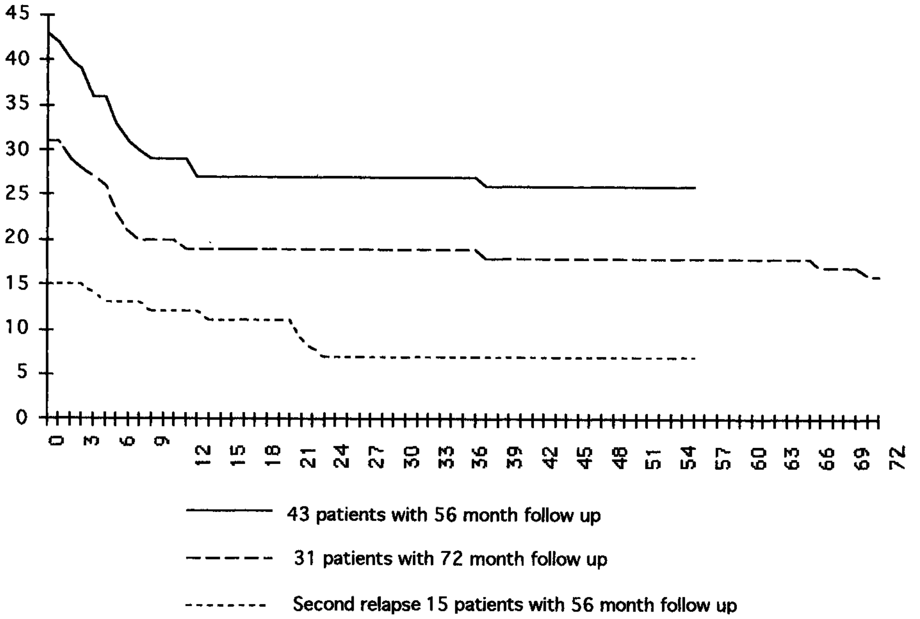

Fig. 1. Survival analysis based on number of months the women were followed from the initial cure.

otics into simple and broad-spectrum antibiotics. When the patient did not remember the antibiotic, we chose to classify her in the broad-spectrum antibiotic group if treatment was given for infection other than urinary tract infection or upper respiratory tract infection. This is not a very precise definition, but the use of simple or broad-spectrum antibiotics did not influence the recurrence rate of BV.

An association has been reported between intrauterine device (IUD) users and BV.[9,13] In our study, IUD use was not associated with relapse of BV, but only 11 women used an IUD.

A long-term follow-up study could show an increased rate of hysterectomies among women with relapse of BV if bleeding disturbances are related to BV.[14,15] We found no correlation between relapse of BV and bleeding disturbances or hysterectomies. However, we found a very high overall gynecological morbidity among the 44 women studied. Fifteen women had had major gynecological operations during the study period, i.e., incontinence (1), laparoscopy (3), hysterectomy (8), and adnexal-tubal operations (3). Four women had had other surgery.

Relapse of BV was not related to the development of CIN, but a high overall incidence of CIN

was present. Nine of the 44 women had CIN diagnosed during the study period, including 3 women with severe CIN or cancer in situ (CIS). This could be a coincidence, a selection bias, or a result of an existing correlation between BV and CIN.[16] Our results suggest that a woman who has ever had BV might be at risk of developing CIN and may even be recommended for regular screening for cytological atypia.

The main weakness of this study is that we have not examined the women on a scheduled basis every year. We therefore do not know if all episodes of BV have come to our attention. However, our population has been rather stable in time and place, which is why it has been possible to investigate most of the women at our outpatient clinic on a regular basis. This study was initiated 5 years after the initial treatment study. Although we had no intention of a follow-up study, most of the patients (33 women) were still visiting our clinic and the follow-up study was easy to perform. The 4 women with self-taken smears had moved to another region in the country a couple of years after the initial treatment study, and our only knowledge of these women came from the questionnaire and the self-taken smear. Three of the 4 women had a typical BV flora on the wet smear at 6 years follow-up and were in the relapse group. The exact time for the relapse is uncertain but is calculated as the day of the diagnosis. As the women with self-taken smears were not diagnosed according to the criteria of Amsel et al.[9] but solely by microscopy, we discussed the possibility of excluding them from further analysis. However, we have shown that diagnosing BV with rehydrated air-dried smears is a reliable alternative to the criteria of Amsel et al. (Kappa index = 0.93 comparing the two methods).[11] An exclusion of these women would lead to a lower relapse rate of BV.

Among pregnant women BV can shift spontaneously to a normal vaginal flora,[17] but this has not been shown for non-pregnant women. If BV is a self-curable disease we would expect the cure rate after placebo treatment to be much higher than the 5–10% cure rates reported after oral placebo.[2] Only one longitudinal follow-up study of untreated BV in non-pregnant women has been published, in which about 30% of the women with BV were cured spontaneously over a 6 month period.[18] However, the diagnosis of BV was not according to the established diagnosing methods for BV, but "vaginal discharge was defined as abnormal and considered suggestive of BV if it was negative for trichomonads and yeast and had one of the following characteristics: 1) excessive volume, 2) abnormal consistency, or 3) characteristic fishy odor."[18] The persistence of BV according to this definition is difficult to compare to established criteria.[9]

The $H_2O_2$ production by lactobacilli may act as an antimicrobial defense system in the vagina. Eschenbach et al.[12] found a low $H_2O_2$ production of lactobacilli from women with BV and Hawes et al.[19] found that $H_2O_2$-producing lactobacilli prevented the acquisition of BV. We cultured all women with lactobacillus flora on wet smear at the 6 years follow-up visit. We were only able to isolate lactobacilli (from Rogosa and blood agar cultured anaerobically) in half of the women, although the wet smears had lactobacillus morphotypes. This could be due to transportation difficulties as the vaginal samples were transported by mail using Stuart's transport medium with transportation time of 1–3 days. We did not find any correlation between the $H_2O_2$ production of the lactobacilli and relapes of BV and we only isolated very few lactobacilli. However, the lactobacilli cultured may not have been the predominating species visible in the wet smear, and our cultures were often performed months or years after the BV recovery.

Survival analysis of the 43 patients with a follow-up time of 56 months shows that most of the relapses occurred during the first year after treatment. In the survival analysis based on 72 months observation two very late relapses occurred. For these two women, we feel quite sure that the relapse is as late as indicated, as both reported that they just developed symptoms of BV and planned to call us for an appointment when the follow-up visit was offered. Survival analysis of 15 women with a second relapse also shows that the second relapse occurred within the first 2 years after treatment, but this observation is not as distinct as for the first relapse.

Data from both Gardner[20] and Amsel et al.[9] suggest a sexual transmission of BV. Gardner[20] inoculated pregnant women with vaginal fluid from women with BV and showed that the inoculated women got BV. In a survey from a health screening program for cytological atypia we found that women with BV significantly more often had had new sexual contacts than women without BV.[21] Hawes et al.[19] reported that the risk of acquiring BV doubled during a 2 year follow-up period if the women had had a new sexual partner. Among monogamous lesbians, BV occurred in both partners 20 times more often if one of the women had BV, suggesting a sexual transmission between lesbians.[22] Our data also support the theory of sexual transmission for BV as a new sexual contact was related to relapses. However, we do not know if the new sexual contact occurred in relation to the relapse. The early relapses may have another etiology than the late relapses where a new infection from a contagious relation may play a role.

On the basis of this study, the relapse rate after successful treatment of BV appears relatively low, especially after the first year, and we recommend treatment of asymptomatic as well as symptomatic women with BV, and stress the importance of careful follow-up.

### REFERENCES

1. Pheifer TA, Forsyth PS, Durfee MA, Pollock HM, Holmes KK: Nonspecific vaginitis: role of *Haemophilus vaginalis* and treatment of metronidazole. N Engl J Med 298:1429–1434, 1978.
2. Larsson PG: Treatment of bacterial vaginosis (editorial). Int J STD AIDS 3:239–247, 1992.
3. Sobel JD, Schmitt C, Meriwether C: Long-term follow-up of patients with bacterial vaginosis treated with oral metronidazole and topical clindamycin (letter). J Infect Dis 167:783–784, 1993.
4. Larsson PG, Bergman B, Forsum U, Påhlson C: Treatment of bacterial vaginosis in women with vaginal bleeding complications or discharge and harboring *Mobiluncus*. Gynecol Obstet Invest 29:296–300, 1990.
5. Hart G: Risk profiles and epidemiologic interrelationships of sexually transmitted diseases. Sex Transm Dis 20:126–136, 1993.
6. Joesoef MR, Wiknjosastro G, Norojono W, et al.: Coinfection with chlamydia and gonorrhoea among pregnant women and bacterial vaginosis. Int J STD AIDS 7:61–64, 1996.
7. Eschenbach DA, Hillier S, Critchlow C, et al.: Diagnosis and clinical manifestations of bacterial vaginosis. Am J Obstet Gynecol 158:819–828, 1988.
8. Thomason JL, Gelbart SM, Anderson RJ, et al.: Statistical evaluation of diagnostic criteria for bacterial vaginosis. Am J Obstet Gynecol 162:155–160, 1990.
9. Amsel R, Totten PA, Spiegel CA, et al.: Nonspecific vaginitis. Diagnostic criteria and microbial and epidemiologic associations. Am J Med 74:14–22, 1983.
10. Morgan DJ, Aboud CJ, McCaffrey IM, et al.: Comparison of Gram-stained smears prepared from blind vaginal swabs with those obtained at speculum examination for the assessment of vaginal flora. Br J Obstet Gynaecol 103:1105–1108, 1996.
11. Larsson PG, Platz-Christensen JJ: Enumeration of clue cells in rehydrated air-dried vaginal wet smears for the diagnosis of bacterial vaginosis. Obstet Gynecol 6:727–730, 1990.
12. Eschenbach DA, Davick PR, Williams BL, et al.: Prevalence of hydrogen peroxide-producing *Lactobacillus* species in normal women and women with bacterial vaginosis. J Clin Microbiol 27:251–256, 1989.
13. Moi H: Prevalence of bacterial vaginosis and its association with genital infections, inflammation and contraceptive methods in women attending sexually transmitted disease and primary health clinics. Int J STD AIDS 1:86–94, 1990.
14. Larsson PG, Bergman BB: Is there a causal connection between motile curved rods, *Mobiluncus* species, and bleeding complications? Am J Obstet Gynecol 154:107–108, 1986.
15. Nygaard OG, Csángó PA, Jagars G: Treatment in general practice of bacterial vaginosis associated with *Mobiluncus* species (English abstract). Tidsskr Nor Lægeforen 111:845–847, 1991.
16. Platz-Christensen JJ, Sundstrom E, Larsson PG: Bacterial vaginosis and cervical intraepithelial neoplasia. Acta Obstet Gynecol Scand 73:586–588, 1994.
17. Platz-Christensen JJ, Pernevi P, Hagmar B, et al.: A longitudinal follow-up of bacterial vaginosis during pregnancy. Acta Obstet Gynecol Scand 72:99–102, 1993.
18. Bump RC, Zuspan FP, Buesching IW, Ayers LW, Stephens TJ: The prevalence, six-month persistence, and predictive values of laboratory indicators of bacterial vaginosis (nonspecific vaginitis) in asymptomatic women. Am J Obstet Gynecol 150:917–924, 1984.
19. Hawes SE, Hillier SL, Benedetti J, et al.: Hydrogen peroxide-producing lactobacilli and acquisition of vaginal infections. J Infect Dis 174:1058–1063, 1996.
20. Gardner HL: *Haemophilus vaginalis* vaginitis after twenty-five years. Am J Obstet Gynecol 137:385–390, 1980.
21. Larsson PG, Platz-Christensen JJ, Sundström E: Is bacterial vaginosis a sexually transmitted disease? Int J STD AIDS 2:362–364, 1991.
22. Berger BJ, Kolton S, Zenilman JM, et al.: Bacterial vaginosis in lesbians: A sexually transmitted disease. Clin Infect Dis 21:1402–1405, 1995.
